# Around the Hospitals

**Published:** 1895-05-04

**Authors:** 


### AROUND THE HOSPITALS.

[Notice to the Managers of Institutions.—Special space is now reserved for the insertion of notices of hospital meetings and festivals. The Editor requests that all notices may reach The Hospital Office, 428, Strand, at least one week before the date of each meeting.]

**London Fever Hospital.**—The annual report of the London Fever Hospital for the year 1894 shows that scarlet fever was less prevalent in London than during the previous year. The mortality was only 2·0 per cent. During the latter part of the year Behring's treatment of diphtheria by anti-toxin was adopted, but the number of cases in which the treatment was carried out was not sufficient to warrant any numerical estimate of its success. Early in the year the new nursing home was completed, and was opened by Lady Balfour of Burleigh in May. The question of rebuilding the hospital has been under consideration by a subcommittee, and the first part of the work, the construction of a new laundry, is to be commenced at once. There was an increase in the annual subscriptions of £140, but on the other hand there was a heavy falling off in donations.

